# Predicting the preferences for involvement in medical decision making among patients with mental disorders

**DOI:** 10.1371/journal.pone.0182203

**Published:** 2017-08-24

**Authors:** Svea Michaelis, Levente Kriston, Martin Härter, Birgit Watzke, Holger Schulz, Hanne Melchior

**Affiliations:** 1 Department of Medical Psychology, University Medical Center Hamburg-Eppendorf, Hamburg, Germany; 2 Department of Psychology – Clinical Psychology and Psychotherapy Research, University of Zürich, Switzerland; KU, UNITED STATES

## Abstract

**Background:**

The involvement of patients in medical decision making has been investigated widely in somatic diseases. However, little is known about the preferences for involvement and variables that could predict these preferences in patients with mental disorders.

**Objective:**

This study aims to determine what roles mentally ill patients actually want to assume when making medical decisions and to identify the variables that could predict this role, including patients’ self-efficacy.

**Method:**

Demographic and clinical data of 798 patients with mental disorders from three psychotherapeutic units in Germany were elicited using self-report questionnaires. Control preference was measured using the Control Preferences Scale, and patients’ perceived self-efficacy was assessed using the Self-Efficacy Scale. Bivariate and multivariate regression analyses were conducted to investigate the associations between patient variables and control preference.

**Results:**

Most patients preferred a collaborative role (57.5%), followed by a semi passive (21.2%), a partly autonomous (16.2%), an autonomous (2.8%) and a fully passive (2.3%) role when making medical decisions. Age, sex, diagnosis, employment status, medical pretreatment and perceived self-efficacy were associated with the preference for involvement in the multivariate logistic model.

**Conclusion:**

Our results confirm the preferences for involvement in medical decisions of mentally ill patients. We reconfirmed previous findings that older patients prefer a shared role over an autonomous role and that subjects with a high qualification prefer a more autonomous role over a shared role. The knowledge about predictors may help strengthen treatment effectiveness because matching the preferred and actual role preferences has been shown to improve clinical outcome.

## Introduction

Patient centeredness has become increasingly important in health care delivery and is justified on both humane [1] and medico-legal grounds [2]. Research activity on it has proliferated in the past several years [1]. Empirical results show that patients wish to be involved in their own healthcare both regarding physical diseases [3] and mental health [4]; however, only a few randomized controlled studies have been published regarding the effectiveness of patient participation [5, 6]. One approach to describe the interaction between a patient and a health care team when making medical decisions, is the model of shared decision making (SDM).

SDM is used when there is no clear ‘right’ or ‘wrong’ treatment, as in the case of equivocal or uncertain evidence of benefit [7]. Physician and patient are considered equal partners who both contribute important information to the decision-making process [8]. Physicians provide professional knowledge and clinical expertise, while the clients best know their personal values and experiences. Both parties go through the process of decision-making together, sharing information and preferences so that the patients are able to evaluate the trade-offs between the advantages and disadvantages of an alternative treatment [9]. Thus, both jointly arrive at a consensus on treatment [7, 10]. This is in contrast to other models, where decisions are made on behalf of only the clinician (paternalistic model [11]) or only the patient (autonomous model [12]).

The extent to which patients wish to be involved in medical decisions may vary depending on the type of disease, medical decision or personal factors [13] e.g. social and cultural factors [14]. One way to measure SDM is to explore patient characteristics that may influence the decision-making process, such as the preference for participation in decision making [15] or, in other words, the preference for involvement.

To date, most researchers have examined the control preferences of patients with cancer or other somatic diseases, and little is known about the control preferences of patients with mental disorders [10]. Mental disorders are highly prevalent and often show a chronic course. There is evidence regarding the effectiveness of different treatment options (e.g., pharmacotherapy and/or psychotherapy [16]), specifically regarding the type of psychotherapy, (e.g., cognitive-behavioral therapy or psychodynamic therapy [17, 18]) or the treatment setting (e.g., outpatient or inpatient treatment). The treatment guidelines for mental disorders underline the importance of involving the patients’ personal factors when making medical decisions (e.g., patient’s preference, patient’s availability, cost of treatment, waiting times for psychotherapy, side effects and drug interactions [19]). Thus, the process of decision making regarding treatment options and patients’ preferences for involvement is highly relevant in the treatment process and outcome [10].Keating et al. [20] and Lantz et al. [21] were able to show that the matching of preferred and actual roles in the decision-making process for breast cancer patients is a strong predictor of patient satisfaction.

Therefore, it is important to identify the patient characteristics that could predict their preferences for participation to offer the right amount of involvement. Some research on the prediction of preference for involvement showed that being female was associated with a wish for greater involvement in decision making [1, 9, 22, 23]. Other researchers did not find an association between gender and preference for involvement [8, 24–27]. Most of these studies investigated patients with chronic somatic diseases like multiple sclerosis, cancer, rheumatoid arthritis or type 2 diabetes. The two patient variables that were found to predict control preference consistently are age [22, 23, 28–43] and education [1, 9, 22, 26, 29, 31, 32, 34–36, 38, 39, 43–45], with younger and higher educated subjects with chronic diseases (e.g., hypertension, diabetes, or breast cancer) preferring an autonomous role. However, O’Neil et al. [46] examined patients with mental disorders and found that older patients preferred greater involvement than younger ones. Incongruent results were also found for race/nationality [13, 23], socio economic status [9, 24] and marital status [9]. Another factor that has been shown to influence control preferences is perceived self-efficacy among psychiatric patients [47].

Perceived self-efficacy is someone’s general belief in one’s own ability to control challenging demands and to achieve a successful outcome by taking an action. It is based on Bandura’s Social Cognitive Theory that states that self-efficacy determines how someone thinks, feels and behaves [48]. Patients with a high perceived self-efficacy believe that their own behaviors influence outcomes. Hence, it is likely to prepare the patient to seek an active involvement. It could therefore determine someone’s control preferences [47].

Although research on patient participation and shared and informed decision making have proliferated within the last few years [2], it has mainly focused on somatic diseases. Regarding mental diseases, current findings by Puschner et al. show that most patients with severe mental illness prefer an active involvement when making medical decisions [49]. However only little is known about the predictors of control preference for patients with common mental disorders. Therefore, the aims of this study were

1. to explore the preferences for involvement of mentally ill patients and
2. to identify the predictive value of patient variables on the preferences for involvement of mentally ill patients. We investigated age, gender, diagnosis, partnership status, educational level, vocational training, employment status, medical pretreatment, symptom severity and self-efficacy as possible predictors.

## Methods

### Study design

This study was part of a research project that investigated the effectiveness of inpatient psychotherapeutic treatment in a prospective multicenter observational study design [50]. Over a period of 22 months, a consecutive sample of patients with mental disorders was recruited.

Inpatient treatment for mental disorders is widespread, with approximately 300.000–400.000 patients treated per year and is thus a relevant part of routine care in Germany[51]. Inpatient treatment includes individual and group psychotherapy. To provide a high external validity, three hospitals with different psychotherapeutic focuses (i.e., cognitive behavioral therapy, psychodynamic focus and an integrative approach) were included, however, all hospitals in this study employed therapists from all therapeutic schools. These hospitals are part of an inpatient setting which is unique in Germany (in German: Psychosomtische Rehabilitation): In contrary to other countries, patients with rather mild and moderately severe mental disorders (mainly F3 –F6 diagnoses, according to the International Statistical Classification of Diseases and Related Health Problems 10^th^ Revision, ICD-10[52]) receive an inpatient treatment with an emphasis on psychotherapeutic interventions within a multimodal treatment approach. In the present study, the treatment consisted of at least one session of individual psychotherapy and two sessions of group psychotherapy per week. Additionally, the patients received psychoeducation, exercise and relaxation training.

The inclusion criteria for patients were a clinical diagnosis of at least one mental disorder according to the ICD-10[52] and an age of 18 years or older. Patients with insufficient German language skills or high cognitive impairment were excluded from the study.

Eligible patients were informed about the study and asked to participate during the first three days after admission. Written informed consent was obtained from all participants. The capacity to consent was checked and proved by the physician or psychologist, who asked the patients to participate.

The study protocol was approved by the human ethics committee of the responsible medical association (the Hamburg Medical Chamber) and complied with the Declaration of Helsinki and the Good Clinical Practice Guidelines.

### Measures

#### Outcome measure: Control preference

To investigate the preference for involvement in medical decisions or the control preferences, we used the German version of the CPS from Degner et al. [53]. The CPS consists of five cards that each present a different role in treatment decision making by using a statement and a cartoon. Patients were asked to sort five possible roles from the most to the least preferred. The five role options are: (A) “I prefer to make medical decisions on my own”, (B) “I prefer to make medical decisions on my own after considering my doctor’s opinion”, (C) “I prefer to make medical decisions together with my doctor”, (D) “I prefer my doctor to make medical decisions for me after seriously considering my opinion”, and (E) “I prefer my doctor to make medical decisions for me”. In the original version, Degner et al. [53] suggested that the card order from the most to the least preferred role needed to be attained by making successive paired comparisons of the cards. We attempted to obtain ratings relevant to clinical practice and, therefore, asked patients for their preference order without making paired comparisons and used only the first choice for analysis.

We conducted the study in an inpatient setting, where the patients do not interact exclusively with the psychotherapist or physician in charge, but also with other staff member of the treatment team. For handling this aspect, we instructed the patients to pick the preferred degree of control they want to get in the interaction solely between themselves and their individual responsible psychotherapist or physician. However, we cannot exclude the possible confusion of influences, if some patients may have referred to other members of the team.

The CPS has been shown to be a reliable and valid instrument [3, 23, 24, 53, 54].

#### Symptom severity

Symptom severity was measured using the HEALTH-49 questionnaire (Hamburger Modules for Measuring Generic Aspects of Psychosocial Health in the Therapeutic Practice) [55]. The HEALTH-49 is a self-report questionnaire that measures different aspects of psychosocial health and consists of 6 modules. Each of these modules can be used independently. To assess generic symptom severity within the last week, we used module A (“psychiatric and somatoform complaints”). It consists of 18 items that have to be rated on a Likert scale ranging from 0 (= not at all) to 4 (= very much), with higher mean scores indicating higher symptom severity. The HEALTH-49 questionnaire has been shown to be comprehensive, broad, generic, economical, and valid and is therefore highly suited for clinical use [55].

#### Self-efficacy

General perceived self-efficacy was measured using the German version of the Self-Efficacy Scale (SES) [56]. With this scale, one’s global self-confidence to handle stressful situations and to achieve a successful outcome by taking an action is measured. The scale consists of 10 items (e.g., “I can always manage to solve difficult problems if I try hard enough”). The items are rated on a Likert scale ranging from 1 (= not at all true) to 4 (= exactly true). The summed score ranges from 10 to 40, with higher scores indicating higher levels of general self-efficacy. The scale has high reliability and construct validity [57]. The internal consistency of our sample was comparable to those reported in the literature (Cronbach’s alpha = .93) [56].

#### Demographic variables and clinical data

Demographic and clinical measures were collected using patient self-report questionnaires. Diagnoses according to the ICD-10 [58] were taken from the physicians’ clinical reports.

### Statistical analysis

To answer the first research question regarding the distribution of the preference for involvement, descriptive statistics were used. To answer the second research question regarding the association between patient characteristics, including the patients’ self-efficacy and the control preference, logistic regression analyses were performed. The outcome was measured with the CPS, which consists of five answer categories. All categorical predictor measures were binary. To examine the bivariate associations between predictors and outcomes, bivariate logistic regression analyses were performed. To examine multivariate associations, multinomial logistic regression analyses were used.

Due to missing values in some variables, especially demographic and clinical characteristics, the dataset for the regression analyses was reduced. To control for the selective effects of the patients that were not included in the model, we compared the included subsample of patients with the drop-out subsample for all tested variables using chi-square and *t*-tests.

All data were analyzed using PASW version 18 statistical software (Chicago, IL, USA).

## Results

### Study sample

Table 1 shows the characteristics of the total sample of 798 patients included in the analysis. The average age was 42.3 years (SD: 11.8; range: 18–77 years), and 77.5% (n = 609) of the patients were women. Most participants (66.1% [n = 454]) were diagnosed with depressive disorders, followed by those with anxiety disorders (19.5% [n = 134]), eating disorders (19.5% [n = 134]), adjustment disorders (17.3% [n = 119]), somatoform disorders (13.4% [n = 92]) or others (23.9% [n = 160]). Half of the sample (54.6% [n = 337]) was in a partnership, and 26.9% (n = 213) had a low educational level (secondary general school or without graduation). Approximately two-thirds were employed (65.4% [n = 522]), and 26.2% (n = 209) of participants had a high vocational qualification (university or college degree). Within the six months prior to admission, approximately half of the sample (51.9% [n = 387]) had received a psychopharmacological pretreatment due to their mental disorder.

**Table 1:** Descriptive statistics for samples with and without empty cells.

	Total sample (n = 798)	Subsample with empty cells (n = 281)	Subsample, no empty cells (n = 517)	Significance test
Demographic and clinical characteristics	n (%) / M (SD)	n (%) / M (SD)	n (%) / M (SD)	t-test / χ^2^-test / FE-test
**Age**				t(782) = -0.564p = .573 d = 0.03
In years	42.3 (11.8)	43.13 (12.6)	42.8 (11.26)	
Range	18–77	18–70	18–77	
**Sex**				χ^2^(1,n = 788) = 0.666p = .414 ϕ = -0.029
Female	609 (77.5%)	214 (79.0%)	387 (76.9%)	
Male	179 (22.5%)	57 (21.0%)	116 (23.1%)	
Diagnosis*				
Depressive disorder (F32, F33 and 34.1)	454 (66.1%)	109 (64.1%)	345 (68.6%)	χ^2^(1,n = 687) = 0.390p = .532 ϕ = -0.024
Anxiety disorder (F40, F41 and F48)	134 (19.5%)	30 (17.6%)	104 (20.7%)	χ^2^(1,n = 687) = 0.497p = .481 ϕ = -0.027
Eating disorder (F50)	134 (19.5%)	40 (23.5%)	94 (18.7%)	χ^2^(1,n = 687) = 2.330p = .127 ϕ = 0.085
Adjustment disorder (F43.2)	119 (17.3%)	26 (15.3%)	93 (18.5%)	χ^2^(1,n = 687) = 0.684p = .421 ϕ = -0.031
Somatoform disorder (F45.1)	92 (13.4%)	24 (14.1%)	68 (13.5%)	χ^2^(1,n = 687) = 0.103p = .749 ϕ = 0.012
Other diagnosis	160 (23.9%)	42 (25.3%)	118 (23.5%)	χ^2^(1,n = 669) = 0.233p = .630 ϕ = 0.019
**Partnership status**				χ^2^(1,n = 773) = 2.566p = .109 ϕ = -0.058
With a partner (married or unmarried)	337 (54.6%)	134 (52.3%)	295 (58.1%)	
Without a partner	436 (42.2%)	122 (47.7%)	208 (41.4%)	
**Educational level**				χ^2^(1,n = 792) = 13.765p < .001 ϕ = -0.132
Low	213 (26.9%)	96 (34.9%)	114 (22.7%)	
High**	579 (73.1%)	179 (65.1%)	389 (77.3%)	
**Vocational qualification**				χ^2^(1,n = 732) = 5.785p = .016 ϕ = -0.089
Low (none, still undergoing training)	523 (65.5%)	167 (77.7%)	346 (68.8%)	
High (college, university)	209 (26.2%)	48 (22.3%)	157 (31.2%)	
**Employment status**				χ^2^(1,n = 707) = 4.743p = .029 ϕ = -0.082
Employed	522 (65.4%)	138 (67.9%)	384 (76.3%)	
Unemployed/other	185 (23.2%)	66 (32.1%)	119 (23.7%)	
**Medical pretreatment** (inpatient or outpatient)				χ^2^(1,n = 793) = 2.651p = .104 ϕ = 0.058
No	359 (48.1%)	121 (43.8%)	245 (48.7%)	
Yes	387 (51.9%)	155 (56.2%)	258 (51.3%)	
**Symptom-severity**				t(790) = -1.595p = .111 d = 0.09
HEALTH-49A	1.44 (0.79)	1.50 (0.82)	1.43 (0.76)	
Range	0.01–3.72	0.06–3.72	0.06–3.72	
**Self-efficacy**				t(787) = 1.820p = .069 d = 0.06
SES score	23.36 (6.7)	22.75 (6.81)	23.51 (6.56)	
Range	10.00–40.00	10.00–40.00	10.00–40.00	
**Control preferences** First preference on the CPS				χ^2^(4,n = 746) = 0.482p = .975 ϕ = 0.025
A- I prefer to make the decision about which treatment I will receive.	21 (2.8%)	6 (2.1%)	15 (2.9%)	
B- I prefer to make the final decision about my treatment after seriously considering my doctor’s opinion.	121 (16.2%)	37 (16.2%)	84 (16.2%)	
C- I prefer that my doctor and I share responsibility for deciding which treatment is best for me.	429 (57.5%)	133 (58.1%)	296 (57.3%)	
D-I prefer that my doctor makes the final decision about which treatment will be used, but seriously considers my opinion.	158 (21.2%)	49 (21.4%)	109 (21.1%)	
E- I prefer to leave all decisions regarding treatment to my doctor.	17 (2.3%)	4 (1.7%)	13 (2.5%)	

*M*: mean; *SD*: standard deviation; χ^2^: chi^2^-test; *p*: p-value; ϕ: effect size phi; *d*: effect size Cohen’s d

*All patients with a depressive disorder as the principal diagnosis and all patients with depression as a co-occurring diagnosis were categorized to “depressive disorders.” This categorization extends to all diagnostic groups

**Graduation (more than nine years of school in the German system)

### Subsample that was excluded in the multivariate analysis (due to empty cells)

Table 1 shows the results of the comparison between the subsample included in the multivariate analysis and the subsample that was excluded. For the variables of age, sex, diagnoses, partnership status, medical pretreatment, self-efficacy, symptom severity and control preferences, no statistically significant differences could be observed between the subsamples. The subsample that was excluded from the multivariate analysis had more participants who had a lower educational level (p < .001), had lower vocational qualifications (p = .016) and were unemployed (p = .029) compared to the subsample that was included. These differences showed statistical significance, but the effect sizes were small (educational level, ϕ = -0.132; vocational qualification, ϕ = -0.089, employment status, ϕ = -0.082). For all of the other tested variables, no statistically significant differences were found.

### Preference for involvement

Table 1 presents the distribution of patients’ decision-making preferences. Approximately 57.5% (n = 429) of all mentally ill patients had a collaborative decision-making preference and wished to share the decision (option C). Furthermore, 21.2% (n = 158) of the patients chose option D, indicating that they wanted their doctor to make the decision after he considered the patient’s opinion, and 16.2% (n = 121) of the patients wanted to make the decision alone after considering the doctor’s opinion (option B). Another 2.8% (n = 21) desired to make the treatment decision on their own (option A), while 2.3% (n = 17) preferred the physician to make the treatment decision (option E).

### Predicting the preference for involvement

The results of the logistic regression analyses with control preference as the dependent variable are shown in Table 2.

**Table 2:** Predictors of various role preferences in comparison with shared role preferences.

	Option A—I prefer to make the decision about which treatment I will receive. ^a^		Option B—I prefer to make the final decision about my treatment after seriously considering my doctor’s opinion.^a^		Option D—I prefer that my doctor makes the final decision about which treatment will be used, but seriously considers my opinion.^a^		Option E—I prefer to leave all decisions regarding treatment to my doctor. ^a^	
	Univariate	Multivariate	Univariate	Multivariate	Univariate	Multivariate	Univariate	Multivariate
Predictor	OR [95%-CI]		OR [95%-CI]		OR [95%-CI]		OR [95%-CI]	
**Age**	0.984 [0.949; 1.021]	0.909 [0.846; 0.977]**	0.997 [0.980; 1.014]	1.008 [0.981; 1.036]	0.999 [0.984; 1.015]	1.005 [0.981; 1.029]	1.016 [0.973; 1.061]	1.004 [0.940; 1.072]
**Sex**								
Female	0.247 [0.101; 0.603]**	0.133 [0.027; 0.658]*	0.531 [0.335; 0.841]**	0.683 [0.365; 1.276]	0.680 [0.439;1.053]°	0.739 [0.415;1.316]	0.731 [0.232; 2.301]	0.736 [0.159; 3.398]
**Diagnosis**								
Depressive disorder	0.270 [0.104;0.703]**	0.138 [0.021; 0.895]*	0.875 [0.556; 1.379]	0.976 [0.408; 2.337]	0.968 [0.636;1.473]	1.079 [0.510; 2.285]	1.018 [0.346; 2.996]	19.613 [1.045; 368.213]*
Anxiety disorder	0.771 [0.219;2.715]	0.264 [0.018; 3.821]	1.125 [0.664; 1.907]	1.601 [0.847; 3.027]	1.018 [0.622; 1.666]	1.121 [0.624; 2.016]	0.948 [0.263; 3.415]	2.134 [0.479; 9.504]
Eating disorder	1.419 [0.496;4.063]	0.321 [0.047; 2.180]	0.913 [0.528; 1.580]	0.943 [0.428; 2.079]	1.077 [0.665;1.744]	1.248 [0.644; 2.420]	0.568 [0.126; 2.552]	1.502 [0.261; 8.651]
Adjustment disorder	2.408 [0.880;6.584]°	0.435 [0.053;3.565]	0.985 [0.548; 1.773]	1.050 [0.364;3.025]	0.899 [0.519; 1.557]	1.057 [0.414; 2.696]	3.130 [1.096;8.937]	30.733 [1.645; 574.262]*
Somatic disorder	1.018 [0.288; 3.606]	2.911 [0.383;22.145]	0.560 [0.276; 1.137]	0.400 [0.158;1.011]°	0.623 [0.335; 1.158]	0.750 [0.375; 1.500]	0.776 [0.172; 3.505]	0.552 [0.064; 4.737]
Others	0.771 [0.248; 2.397]	0.635 [0.095; 4.227]	0.539 [0.305; 0.953]*	0.508 [0.254;0.018]°	0.828 [0.521; 1.316]	0.569 [0.313; 1.034]°	0.899 [0.283; 2.853]	0.456 [0.073; 2.862]
**Partnership status**								
With a partner	0.701 [0.291; 1.687]	0.236 [0.050; 1.117]°	1.086 [0.718; 1.643]	0.865 [0.506;1.478]	1.001 [0.690; 1.454]	0.925 [0.575; 1.488]	0.600 [0.219; 1.641]	0.433 [0.126; 1.487]
**Educational level**								
High^b^	0.652 [0.256; 1.659]	1.387 [0.278; 6.921]	1.375 [0.830; 2.279]	1.340 [0.637;2.820]	0.794 [0.528; 1.194]	0.815 [0.451; 1.470]	0.598 [0.216; 1.656]	0.503 [0.119; 2.129]
**Vocational qualification**								
High^c^	0.641 [0.210; 1.958]	0.399 [0.070; 2.285]	1.830 [1.178;2.843]**	1.670 [0.935;2.985]°	0.949 [0.621; 1.449]	0.960 [0.554; 1.663]	0.394 [0.088; 1.775]	0.465 [0.087; 2.491]
**Employment status**								
Employed	0.521 [0.204; 1.332]	0.109 [0.022; 0.540]**	1.072 [0.664; 1.731]	0.862 [0.437; 1.699]	1.279 [0.813; 2.014]	1.507 [0.814;2.791]	5.686 [0.742;43.583]°	1.153E8 [1.153E8; 1.153E8]
**Medical pretreatment**	0.204 [0.067; 0.615]**	0.090 [0.014; 0.593]*	0.851 [0.568; 1.274]	1.183 [0.702; 1.996]	1.033 [0.717; 1.490]	1.385 [0.866; 2.213]	0.606 [0.226; 1.621]	0.671 [0.192; 2.339]
**Symptom-severity**: HEALTH-49A	0.488 [0.257; 0.926]*	0.548 [0.166; 1.807]	0.697 [0.531; 0.914]**	0.854 [0.551;1.324]	1.010 [0.802; 1.274]	0.802 [0.552; 1.166]	1.285 [0.710; 2.326]	1.348 [0.532; 3.419]
**Self-efficacy**: SES	1.112 [1.038;1.190]**	1.212 [1.057;1.388]**	1.035 [1.004;1.067]*	0.998 [0.951; 1.049]	0.983 [0.956; 1.011]	0.959 [0.919; 1.002]°	0.985 [0.914; 1.061]	0.941 [0.841; 1.053]

^a^: the reference category is option C “shared decision making”

^b^: Graduation (more than nine years of school in the German system)

^c^: college, university

OR = Odds Ratio; CI = Confidence Interval; the thresholds for significance are (p): .10 (°), .05 (*), .01 (**) and .001 (***)

Information about the general model: p < .001; pseudo-R^2^ = .002; chi^2^ = 112.174

In the bivariate analysis, when not controlling for other variables, sex, diagnosis, vocational training, medical pretreatment, symptom severity and self-efficacy attained statistical significance. Women (OR: 0.247 [0.101;0.603]), patients who were diagnosed with depressive disorder (OR: 0.207 [0.104;0.703]), patients who had undergone medical pretreatment (OR: 0.204 [0.067;0.593]), patients with high symptom severity (OR: 0.488 [0.257;0.926]) and patients with low self-efficacy scores (OR: 1.112 [1.038;1.109]) were less likely to choose an autonomous role over shared decision making. Women were, compared with men, less likely to choose a moderately autonomous role over a shared role (OR: 0.531 [0.335;0.841]). Patients with high vocational qualifications (OR: 1.830 [1.178;2.843]), those with low symptom severity (OR: 0.687 [0.531;0.914]) and those with high self-efficacy ratings (OR: 1.035 [1.004;1.067]) were more likely to choose a moderately autonomous role over a shared role.

As it was the most preferred option, the preference role for shared decision making (option C) was set as the reference group for the regression analyses. In total, 514 patients were included in the model.

In the multivariate analysis, when controlling for other variables, age, gender, a depressive disorder diagnosis, an adjustment disorder diagnosis, employment status, medical pretreatment and self-efficacy attained statistical significance.

For the autonomous preference (option A: “I prefer to make the decision about which treatment I will receive”), age, sex, a depressive disorder diagnosis, employment status, medical pretreatment and self-efficacy attained statistical significance. Men were more likely to choose role A over role C than women (OR for being female: 0.133 [0.027;0.658]). In other words, men were more likely to prefer an autonomous role over a shared role compared with women. Patients who were diagnosed with depressive disorder were more likely to choose a shared role over an autonomous role (OR for diagnosed with a depressive disorder: 0.138 [0.021;0.895]). Older patients were significantly less likely to choose an autonomous role (OR: 0.909 [0.846;0.977]). Subjects who were employed did not prefer autonomous involvement (OR: 0.109 [0.022;0.540]). We observed the same results for patients who underwent medical pretreatment; they did not choose an autonomous role (OR: 0.090 [0.014;0.593]). Patients with a higher rating on the SES preferred autonomous involvement (OR: 1.212 [1.057;1.388]).

No statistically significant relationships were found for the groups that chose either option B, “I prefer that my doctor makes the final decision about which treatment will be used but seriously considers my opinion” or option D, “I prefer my doctor to make medical decisions for me after seriously considering my opinion” as their preferred involvement.

For the passive group (option E: “I prefer to leave all decisions regarding treatment to my doctor”), depression and adjustment disorders attained statistical significance in the multivariate model. Subjects who were diagnosed with depression were more likely to prefer a passive role (OR: 19.613 [1.045;386.213]). Furthermore, patients who were diagnosed with an adjustment disorder chose a passive role (OR: 30.733 [1.645;574.262]).

The explained variance of the total regression model was 0.2% (Pseudo-R² = .002).

## Discussion and conclusion

### Discussion

Not all patients want to be involved into decisions concerning their own health care, and the extent and type of control preferences vary [35]. The main goals of this study were to investigate the control preferences of patients with mental disorders and to identify the patient characteristics that predict them. Our results are partly congruent with previous results and give new insight into this topic.

We found that most of the patients with mental disorders preferred a collaborative role when making medical decisions. These findings confirm numerous results that were found in other studies within the last few years [3, 26, 31]. However, most of these studies examined patients with somatic diseases, mainly cancer [7], and only a little research has been performed on the preference for involvement of mentally ill patients [10]. This could be attributed to prejudices toward possible complications when applying SDM to patients with mental disorders. It is important to know about the preferences of patients with mental disorders to be able to build up a good relationship. Puschner et al. [49] examined the association between clinical decision making and outcome in an observational study with mentally ill patients from different European countries. The results showed that an active involvement in clinical decision making led to a decrease of unmet needs over time. This is important since unmet needs are associated with relevant process and outcome variables such as the therapeutic alliance and quality of life [49].

As long as the patient’s decision-making ability is not limited due to a decisional incapacity [12, 59], a situation where they are at risk to self-harm or an acute psychotic state [7], it is important to elicit the control preferences of mentally ill patients.

We identified variables that could predict control preferences. We were able to confirm findings that suggested that gender is associated with decision making. In the past, research has shown that being female was a predictor of a shared role preference [1, 9, 22, 23, 60]. These findings are congruent with our results, where being female predicted a preference for a shared involvement, while men were more likely to prefer an autonomous style in decision making.

Having undergone medical pretreatment was a predictor of choosing a shared role over an autonomous role. Experiences in a long-lasting psychotherapeutic treatment could lead to a growth in confidence in the treating team [61]. The patient is then able to trust the physician enough to share the control in medical decisions. This assumption, however, questions the statement that control preference is a trait that does not change over time. When Degner et al. [53] established the CPS, they assumed that control preference was an intrinsic personality trait. Different authors have followed this assumption [23]. The results of studies that elicited a control preference at different points in time confirmed this concept [62]. However, it has been discussed that a control preference represents a state rather than a trait of a patient [63]. If a control preference is state, then it is likely to change throughout an illness. Due to the cross-sectional design of our study, we could not investigate this issue.

With regards to age, we were able to replicate the association found by several other studies that younger patients have a stronger desire to decide alone than older patients [23–25, 28, 30, 32]. In the multivariate analyses, we found that older patients were less likely than younger patients to choose an autonomous role over a shared role.

In other studies, educational level was a strong predictor of control preference, but we could not confirm this association. There could be several explanations for this incongruence. Educational level may be a strong predictor of control preference in somatic diseases, the topic most other studies evaluated but not in mental health. We performed a bivariate analysis in addition to the multivariate analysis to compare our results to those of other studies that solely assessed the bivariate relationship between control preference and patient variables. The studies that found that educational level was a predictor of control preference only conducted a bivariate analysis [42–45]. However, our findings indicate that the impact of educational level on control preference might be confounded by other variables (e.g., employment status or vocational training). This explanation is underlined by other results; an association was found between educational level and control preference in the bivariate analysis but not in the multivariate analysis [29, 32]. Employment status and vocational qualification could have a much greater influence on control preference than would the educational level because the former two represent a patient’s current socioeconomic status.

Patients who were diagnosed with a depressive disorder were less likely to prefer an autonomous role and more likely to wish for a passive role in decision-making. This might be due to the typical depressive symptoms. Depression can influence patient’s mood (such as persistent sadness or low mood, loss of interest or pleasure and fatigue), their physical condition (disturbed sleep, low energy, agitation or slowing movements, poor concentration, indecisiveness, poor or increased appetite and libido disorders) and their self-esteem (guilt, self-blame, low self-confidence) [18]. Rumination and indecisiveness are among the main symptoms of depression. It is comprehensible that especially people with these symptoms show a reduced decisional capacity and are therefore not likely to take an active role in the decision-making progress. Similar problems account for the diagnosis of an adjustment disorder. The symptoms (such as sadness, anxiety, nervousness, difficulties concentrating, feeling overwhelmed, desperation and worry) that influence the mood and physical condition of patients can also influence their attitudes toward their own participation and could lead to a reduced decisional capacity.

We focused on examining self-efficacy because we presumed that SE influences control-preference and only a little research has been conducted on this topic. Our results show that self-efficacy was a predictor of preferences regarding the keeping, sharing or giving away of control. As expected, patients with high ratings on the SES were more likely to prefer to make medical decisions alone. Self-efficacy is defined as someone’s belief in one’s own ability to achieve a successful outcome by taking an action [48]. Therefore, self-efficacy is likely to describe a fundamental basis for the desire for an autonomous role in decision making.

However, it has to be considered that the explained variance of the total regression model predicting patients’ preference for involvement was rather small. Future research might focus on further potential predicting variables, in order to get a better understanding of the determinants of control preference.

Furthermore, it needs to be pointed out that research on predicting variables for patient participation cannot replace discussions between the clinician and the patient about the preferred degree of participation. There is evidence that physicians are not able to predict patients’ control preference based on personal variables and the control preference is influenced by the complexity of the physician-patient communication [64]. The duration of the consultation [65], and the communication style and specific interventions are associated with patients’ control preference and involvement [66, 67]. Therefore, clinicians should inquire the patients’ preference for involvement in decision making.

There are some methodological issues of this study that need to be taken into consideration. First, when assessing control preference with the CPS, we did not ask the subjects to consider one particular decision. O’Neil et al. [46] and Patel et al. [13] showed that the control preference depends on the type of decision, and it is therefore important to define one particular decision as a reference. Because all of the patients were at starting an inpatient psychotherapeutic treatment, we assumed that they had just made similar decisions in the past (e.g., to start an inpatient psychotherapeutic treatment). We expected the patients to think of a similar decision and, therefore, did not specify the type of medical decision. However, it is possible, that the patients thought of different decisions when they stated their preferred role. For future research regarding the control preference of mentally ill patients, it might be helpful to name a certain type of decision and the specific clinician to better understand what the preference is about.

Moreover, regarding the distribution of the different control preferences according to the CPS and the interpretation of the prediction model, it should be noticed that the findings on the extreme response options are rather imprecise due to the low number of participants choosing them.

Only 65% of our sample could be included in the regression analysis due to the case-wise exclusion by scattered missing values. Although some of the differences between cases with and without empty cells were statistically significant (without a correction for multiple testing), we considered their practical and clinical relevance as being low, as indicated, for example, by the very small standardized effect sizes. Furthermore, we had no plausible theoretical reasons to assume that the mechanisms leading to missing values in the investigated predictors are associated with the outcome. Consequently, we concluded that missing values can be treated as missing completely at random (MCAR). A complete case analysis was conducted under the MCAR assumption. Nonetheless, a certain unknown bias cannot be ruled out.

Although the setting our study was set in is unique to Germany, with inpatient treatment of patients with common mental disorders, it represents about one third of all patients with mental disorders, treated in an inpatient setting in Germany [51], and thus, is part of the routine inpatient care. With an almost unselected sample of inpatients who were diagnosed with a variety of mental disorders (mainly F3—F6 diagnoses) and were treated by real-world therapists using real-world treatments, the external validity of the current findings is strengthened. However, the perspective of routine mental health care implies some limitations, especially in terms of internal validity. Due to the naturalistic design, we cannot exclude the possibility that variables other than the ones currently assessed are associated with the control preference. Further research should focus on additional covariates that could influence decision-making preferences.

Moreover, this specific setting is only partly comparable to other inpatient settings like inpatient psychiatric treatment (with treatment of other mental disorders, e.g., psychoses). This limits the representativeness of our results to inpatient treatment in this specific setting with mainly psychotherapeutic treatment of common mental disorders. However, we aimed to achieve a high clinical representativeness from our study results based on several aspects of the study design. First, the naturalistic study design and the consecutive inclusion of patients in the study provided a representative image of the real-life, routine inpatient treatment of mental disorders. Second, due to the large and heterogeneous sample, we were able to examine the predictive value of different patient characteristics and control for confounding variables by using a multivariate analysis. Third, we conducted the multicenter study in three psychotherapeutic units to reduce the effects that result from the specific context of a single unit.

### Conclusion

Our results suggest clinically relevant conclusions. First, patients with mental disorders vary in the extent of control preference, but some patients, such as those with somatic diseases, prefer a shared role. Second, there are certain patient characteristics that are associated with the preferences of mentally ill patients. Women, older patients, patients with depressive or adjustment disorders, employed patients and patients with medical pretreatment were less likely to prefer an autonomous role compared with a shared role. On the other hand, patients with high self-efficacy are more likely to prefer an autonomous role rather than a shared role, when considering all other variables. It is important to know about these variables because a matching of preferred and actual role preference has been shown to lead to greater satisfaction and an improvement in clinical outcomes [68]. When working therapeutically with patients who have mental disorders, it is very important to provide a faithful and strong working alliance between the patient and physician or therapist to achieve a good treatment outcome. This seems to be more easily achieved when the patient’s preference is included in the process of medical decision making.
